# The Actual Process of Nutrition and Inflammation in the Living Structure, Demonstrated by the Microscope

**Published:** 1845-07

**Authors:** 


### ART. IX.

*The Actual Process of Nutrition and Inflammation in the Living Structure, demonstrated by the Microscope.* PART II. By WILLIAM ADDISON, F.L.S. &c.—*London,* 1845. 8vo, pp. 114. With Two Plates.

MR. ADDISON'S industry as an observer has furnished in this memoir a large number of new facts, corroborative of his former statements; and our estimate of their value, joined to our respect for himself, induce us to bring them again under the notice of our readers. But we feel obliged to state, at the same time, that our conviction as to the fundamental errors of his reasoning upon these facts, remains unchanged; and that, if such reasoning be admitted in physiology, it can never, in our apprehension, attain the rank of an exact science. Hence we feel bound to put our readers on their guard against these errors; by entering into a more detailed examination of them than we should deem necessary, if they were less connected with important truths, or came forth to the world with less pretension.

We believe that he is fully justified in stating as he does, that "the accumulation of the colourless cells of blood in irritated and inflamed textures, and the existence of active molecules in the interior of the cells of blood, saliva, and pus, have become established physiological facts." But we cannot regard this or any similar fact as "establishing the identity of the cells of mucus, pus, and lymph, with the colourless cells circulating in the blood;" any more than it establishes their identity with those numerous cells of other kinds, which exhibit similar phenomena. And although Mr. Addison expresses himself (p. 13) as "at a loss to understand what amount of evidence upon this point will be considered satisfactory," if this identity be not admitted upon the grounds he has stated, we cannot see how any unprejudiced physiologist, who is acquainted with the general doctrines of cell-life, can feel any difficulty in the matter. The evidence required is simply, that Mr. Addison should trace the passage of a colourless corpuscle of the blood, through the parietes of the blood-vessels, through the intervening tissue, and through the basement-membrane of the mucous surface, before he expects the world to believe that a cell previously floating in the circulating fluid is cast off as an epithelium-cell from the excreting surface.

The first series of facts (chapter I) relates to the existence of *moving molecules* in the interior of cells. These have been observed by Mr. Addison in the colourless cells of the blood, taken from a swelled leg, and from the red patches of erythema nodosum (in which there were nearly as many colourless as red corpuscles;) in the similar cells of a large bulla, resembling that of pemphigus; in the cells contained in a transparent fluid, which oozed from the red shining surface of a nævus on the forehead, that had been irritated by stimulating applications; in the lymph-and pus-cells of herpes labialis; in the pus-cells of porrigo scutellata; in the blood and pus of an ulcerated leg; in the cells of a muco-purulent vaginal discharge; in the pus-cells of the eruption produced by tartar-emetic ointment; and in the cells of the saliva and nasal mucus. The following observation has an interesting relation to Mr. Addison's former statements, with regard to the origin of the fibrinous network of the blood in the contents of its colourless cells. It relates to the bulla just referred to; the covering of which was tense, thin, and shining, without any redness in the surrounding integument.

"I punctured the vesicle with a point of a lancet; and instead of its discharging its contents and collapsing, as I expected, only a small drop of perfectly transparent and limpid fluid flowed out. In this fluid I found several corpuscles or cells; some were evidently red blood-cells; others resembled, and in my opinion were, colourless blood-cells. There were, besides, sundry other forms of cells, some entire, others flattened, and altered in form, having numerous filaments of the utmost degree of tenuity attached to them. All the colourless cells were more or less entirely filled with the most minute molecules; and there were several little masses and congregated groups of isolated molecules attached to each other by long and delicate filaments. Some of the cells were actually discharging molecules; and others had filaments so attached to them, as to appear as if clothed with cilia. There were also several fibrillated networks, composed of filaments, molecules, and corpuscles; and here and there perfect but small epithelial-cells. I now snipped off the point of the vesicle with a pair of scissors, and yet it did not discharge the whole of its fluid. This was explained, when on examining the portion removed by the microscope, it was found to consist of a most intricate network of fibrinous filaments, which ramified through the whole interior of the vesicle, and retained the fluid in its meshes." (p. 5.)

In chapter II we find some observations "On the transformation of pus-cells into a mucous or fibrous tissue, and tubercle; on the fibrous tissue of saliva; and on the coagulation of the blood." These confirm Mr. Addison's former statements as to the rupture of the pus-cells by liquor potassæ, and the consequent formation of a consistent coagulum, which, when treated by dilute acetic acid, assumes the characters of a distinct and coherent fibrous membrane. "The fibrous tissue here formed by my manipulations," he says, (p. 17,) "could not, by any visible or microscopical character be distinguished from that formed by the fibrillation of the buffy layer of the blood, or by the process of nutrition in the living body." We must take leave to express a doubt, however, whether the fibrous appearance was not partly due to the manipulations; which, as every microscopist knows, may give a fibrous appearance to a perfectly homogeneous structure. The following are Mr. Addison's inferences from this fact.

"It is evident that, in this experiment, the plasticity of the resulting material arises from the rupture of the cells. The fluid element of pus, before this event, is limpid; that is, it has no plastic quality or tenacity whatever; it drops from one vessel into another like water; but when a majority of the cells have been ruptured, and their contents mingled with the previously existing fluid, then the whole becomes extremely plastic and coherent; it will no longer drop from one vessel to another, and it exhibits all the microscopical appearances of fibrous tissue or of mucus. The event, therefore, accompanying or preceding this transformation, is the rupture of the pus cells; and the result is a mucous or fibrous tissue." (p. 18.)

It is upon these and similar facts, that Mr. Addison founds his argument as to the *identity* of pus-cells with the white corpuscles of the blood. But they appear to us far from warranting the assumption. According to Mr. Addison's own showing, the white corpuscles of the blood and lymph burst, and emit their coagulable fluid, *of their own accord;* whilst the pus-cells do this only when under the influence of liquor potassæ. Now that the liquor potassæ in this case acts, not only in causing the rupture of the cells (which seems to be Mr. Addison's doctrine,) but also in altering the chemical and physical properties of their contents, must be admitted, we think, to be a far from improbable supposition. At any rate, the contrary has not been proved; and this argument for identity, therefore, completely falls to the ground.

In another experiment, Mr. Addison describes the formation, by the admixture of a few drops of pus and of liquor potassæ, and by subsequent manipulation, of a "delicate, thin transparent, and highly-elastic fibrous membrane, exactly resembling some of the thin transparent membranes of the embryo, except in the presence of blood-vessels, or the structureless basement-membrane of Mr. Bowman." Further on, we find, that if saliva be treated with dilute acetic acid, the coagulum exhibits a distinct and well-defined fibrous structure, (in regard to which, however, we must make the same remark as before;) so that every drop of saliva contains a form of plastic matter, the existence of which may be made visible by dilute acetic acid; doubtless on account of some chemical change which that agent produces in it.

By submitting the fibrous coagula of pus, obtained through the operation of liquor potassæ, to the prolonged agency of dilute acetic acid, they were changed into an "opaque, white, friable substance, resembling tubercle." The same result was obtained when the pus was first diluted with blood-serum; the first result of the treatment by liquor potassæ was to form a transparent plastic mass, which became white and opaque when immersed in dilute acetic acid; this having been washed well in water, and rolled on blotting paper to remove superfluous moisture, its weight was 14½ grains, (being that of the pus + the albuminous constituent of the serum employed;) but moisture continued exuding from it, and at the end of three hours it weighed 10 grains. "A small portion, examined by the microscope, exhibited numerous pus-cells still entire, amorphous granular matter, and myriads of molecules; it had the physical properties, visible appearance, and microscopical character of pulmonary tubercles." (p. 28.) These experiments are additional proofs of the close relation which subsists between the plastic element of the blood and the elements of pus and tubercle; but we do not see that they add much to our knowledge of the exact nature of that relation, since we have no reason to think that the process of transformation, by the agency of liquor potassæ and acetic acid, is *identical* with that which takes place in the living body, but only that it bears a certain degree of *analogy* to it.

In the following conclusions, deduced by Mr. Addison from his experiments, we believe that we may generally concur:

"It appears to me, then, to be demonstrable from my experiments, that the colourless elements or cells of the blood spontaneously transform themselves into an elastic fibrous tissue, after their separation from the living structure; into a plastic transparent mucus (another form of fibrous tissue,) when treated with liquor potassæ; and into flocculi, flakes, and tubercle-like matter, when acted on by water or acetic acid.

"It appears to me also to be demonstrable from my experiments, that a similar fibrous tissue (?) and tubercular matter may be formed by the disintegration of pus-cells, and by treating the saliva with acetic acid or alcohol.

"The fibrous tissues from these sources appear to have mechanical properties, a physical character and texture, a microscopical or visible appearance, and a chemical composition, so closely allied to the fibrous tissues and membranes formed by the process of nutrition in the living structure, as to leave no reasonable ground for doubting, that the latter result from the transformation or disintegration of cells. "If so, then it appears to me to follow as an inevitable conclusion, that there is no such thing in the living organism as a membrane secreting mucus, (taking the ordinary meaning attached to the word secreting;) no such thing as an expanse of fibrous or any other tissue so changing the nature or character of fluids as they filter or transude through its fabric, that the fluid of the blood on one side becomes mucus by merely passing through it to the other; on the contrary, it appears to me that we can no longer hesitate to admit, that mucus, whether conformable to the type of a normal nutrition, or departing from it so as to constitute an abnormal or diseased element, can exist only in virtue of the life of the cells." (p. 38.)

We quote these conclusions, not as being particularly new, (for they have been, in more general terms, set forth in our own pages,) but because they are expressed by Mr. Addison with much clearness and force, as independent deductions from his own experiments. We need not, however, go along with him in his subsequent inquiry, as to whether the secreting cells are "generated in the tissue, or are ulterior forms of blood-cells;" because he adduces no new facts in support of his position, which we have already shown to be quite unsupported by his own observations, and irreconcileable with those of others. We may concisely state Mr. Addison's views to be, that the colourless corpuscles of the blood are the sole agents in nutrition and secretion; that in normal nutrition, the colourless blood-corpuscles adhere to the tissue forming the boundary of the blood-channels; that they pass into and contribute to form the tissue (the parietes of the capillaries;) that they are afterwards evolved or thrown off from the nearest free surface, a follicle, crypt, or duct; and that the epithelial scales and the mucus, or the secretions flowing from the follicles or ducts, are the result of the dissolution of the cells and tissues. We have pointed out on a former occasion, the complete absence of the *kind* of evidence, required to support a proposition so extraordinary, as the passage of any one floating cell, (let alone a vast multitude,) from the interior of the blood-channel to even the nearest mucous surface; opposed as such a passage is, by the septum formed by the basement membrane, which has certainly no apertures in it large enough to allow these cells to pass, and which is certainly not itself formed of coalesced cells about to resolve themselves into epithelium. Moreover, on the surface of this membrane, and within the extremities of glandular follicles, the progressive development of cells from nuclei has been distinctly traced; proving that the cells do not come forth from the subjacent tissues already formed, but are developed in the very part, from which they are afterwards to be thrown out. We might advert to numerous other arguments in opposition to Mr. Addison's views; but many of these are so palpable, that we shall not do our readers the injustice to suppose that they require to have them suggested. Mr. Addison rejects, as unfounded dogmata, the assertions of those, who state that the cells of pus, fibrinous exudations, &c., are formed in the fluids *after* it has left the blood-vessels; preferring to think that they have passed *bodily* out of the vessels, to the surface on which they are found. Now surely the *onus probandi* rests with Mr. Addison; since all that we *know* of the nature of the walls of the blood-vessels opposes the supposition which he has formed, in regard to the constant interchange of full-grown cells between their interior and exterior surfaces.

We think it not so improbable, however, that there *is* such an interchange in regard to the *cell-germs*, or *molecules*, which are set free by the rupture of the white corpuscles. The membrane lining the blood-vessels has been seen, in many instances, to be composed of *coalesced granules;* and granular spots have been seen in the basement-membrane of mucous surfaces and follicles, apparently furnishing the germs from which the epithelium-cells are formed. Our notion of the act of nutrition and secretion, therefore, admits the possibility, that the pus and epithelium-cells, as well as the cells of the solid tissues, may have had the colourless corpuscles for their *parents;* although we do not as yet see any distinct proof of the fact.

In the third chapter of Mr. Addison's Researches, "The structure and functions of the kidney" are professedly considered, with reference to the nature of the fluid element of blood; Mr. Bowman's recent anatomical investigations being taken as the groundwork of his views, We have been able to find very little in it, however, respecting the functions of the kidney; in fact Mr. Addison's views with regard to the relative *products* of the Malpighian capillaries, and of those which surround the uriniferous tubes, appear to us to correspond precisely with those first suggested by Mr. Bowman, and received by other physiologists. But he seems to consider the fact, that a watery fluid, containing little else than saline matter, is filtered off (as it were) through the capillaries of the Malpighian tufts, as a demonstration of his position, that the *fluid* element of the circulating blood does not contain either fibrine or albumen, but consists merely of water holding the saline matter in solution. He argues that we have no reason to regard the walls of these vessels as having the power of *selection*, allowing some of the saline matter of the blood to pass, but keeping back the organic substances also dissolved in it; this power of selection being an attribute of cells only. He does not seem to be aware, however, that recent experiments have shown that dead animal membranes, such as the pleura and peritoneum, *do* possess this very power; separating, as it were, the serum of the blood into its two constituents, saline and albuminous fluids, by allowing the former to pass, whilst the latter is kept back. Hence Mr. Addison's argument falls entirely to the ground.

This chapter contains, a number of other arguments bearing on the same subject; in which, however, we find little that is new, and still less that is good. Mr. Addison objects to some of our former criticisms upon his reasoning; but we do not see that he invalidates the facts upon which our objections were based. For instance, we objected to his drawing inferences from the process of nutrition in fœtal structures, because there are in them (as in the lowest forms of animal life) no proper vessels, but only intercellular passages; to which Mr. Addison contents himself with replying, that as "some of the best energies of physiologists have been occupied in tracing out and establishing a wonderful analogy in the elementary tissues and mode of growth of nutrition in all living structures," we propose to depart from those general principles, by "*assuming* differences, where most probably, if the analogies in all living structures be true, there are none." Now we take leave to think, that we are as competent to judge of the real nature of these analogies as Mr. Addison; our chief attention, for many years past, having been given to the search for them; and we venture to say that there is no analogy that can upset the *fact*, upon which we rested our objection, that there is an important difference between embryonic and adult structures, in regard to the relation between the solids and fluids, which must prevent us from applying to the latter all the inferences that may be founded upon the former. Mr. Addison can scarcely deny, that in the lowest plants and animals, as in the early embryonic condition of the higher, no vessels at all exist; and that there must therefore be in *them* a condition of the nutritive function essentially distinct from that under which this function takes place in the higher adult structures, although the function itself remains fundamentally the same. Again, if the early condition of the blood-channels be mere intercellular passages, not lined by a membrane (a *fact* which it is for Mr. Addison to disprove, if he can), it is reasonable to suppose that there may be conditions of the nutritive action in the tissues which they permeate, different from those which prevail in the adult tissues, in which the capillaries have distinct membranous walls; and that the fact, if fact it be, of the incorporation of the corpuscles floating in the blood, with the tissues in absolute contact with the fluid, does not justify the assumption that such incorporation can take place, when the blood and the tissues are separated by a membrane, that seems totally impermeable to any particles of appreciable size. We are not prepared to deny the *possibility*, that in the *embryonic* condition of tissues, the floating cells of the blood *may* become withdrawn from the blood, and incorporated with the solids; but *if* this takes place, it can be only so long as these solid tissues are themselves evidently composed of cells, or of simple transformations of them; and when they have departed more widely from that type, and the capillaries have acquired distinct membranous walls, we take leave to think that the passage of bodies so large as the colourless corpuscles through those walls is a phenomenon of not very likely occurrence. At any rate, we do not find a single observation of Mr. Addison's, which is not perfectly reconcileable with our own view, that it is the *contents* of the colourless corpuscles, not those bodies themselves, which find their way through the parietes of the vessels.

The last chapter, although entitled a "Demonstration of the phenomena and results of Inflammation," is very far from being such; its account of the process being very imperfect, and being as far from deserving the character of a *demonstration,* as is the account of the process of normal nutrition, which we have already criticised. We have so recently expressed our views on this subject, that we need not here go over the ground again; particularly as we find nothing in Mr. Addison's *observations* that is not in perfect harmony with them, although his *inferences* are widely different from ours. Here and elsewhere he disputes our position, that an increased production of colourless corpuscles takes place in the circulating current during the inflammatory action; and thinks that the large amount seen in the irritated vessels of a frog's foot is due, not to any additional number in the entire mass of blood, but to the determination of those existing in the whole current towards the particular spot. Now this may be, and very probably is, the fact in the case in question; but we grounded our statement, not upon the large proportion of white corpuscles in the vessels of an *inflamed* part, but in the great increase in the whole mass of the circulating fluid,—provided the inflammation has been sufficiently acute and extensive,—as proved by their unusual abundance in blood drawn from a *remote* part. We do not think that Mr. Addison can reason fairly on this point, from the phenomena of inflammation in cold-blooded animals; since it is well known, that this process rarely if ever presents itself in these, in its most acute form,—the suppurative. Mr. Addison thinks that all the colourless corpuscles in the blood are directly furnished by the chyle; and seems to take no account of the molecules, which are set free by their rupture. Now the increase we have just alluded to, seems to prove the fact of their self-generating power; the existence of which we might have inferred from their character as *cells.* And it cannot be thought unreasonable to infer, that this power should be exercised as it is in the analogous cells of the lower cryptogamia; which propagate by the rupture of the parent, and the development of the contained granules into cells. Various recent observations, especially those of Mr. Macleod (see Br. and For. Med. Rev. vol. XIX, p. 565), indicate the existence of a similar process in the cell-development of animals; and although we may freely admit that no one has yet watched the development of cells in an exuded blastema, and that the process is somewhat hypothetical, yet we think our readers will agree with us in regarding Mr. Addison's notion of the passage of the white corpuscles themselves into the exudation, as still more hypothetical. The former view is supported by many analogies, —e.g. the development of the epidermic cells, which *has been* studied. The latter appears to us plainly contradicted by known facts.

We now again take leave of Mr. Addison; thanking him most heartily, in the name of the profession, and in our own, for the large number of valuable facts which he has contributed; but strongly urging him to reconsider his *inferences,* which, so far as we know, have not been thought worthy of adoption by a single Physiologist of any note.

---

